# Exosome-mediated transfer of miR-10b promotes cell invasion in breast cancer

**DOI:** 10.1186/1476-4598-13-256

**Published:** 2014-11-26

**Authors:** Ramesh Singh, Radhika Pochampally, Kounosuke Watabe, Zhaohui Lu, Yin-Yuan Mo

**Affiliations:** Department of Biochemistry and Cancer Institute, University of Mississippi Medical Center, Jackson, MS USA; Department of Microbiology and Cancer Institute, University of Mississippi Medical Center, Jackson, MS USA; Department of Endocrinology, PLA General Hospital, Beijing, 100853 PR China; Department of Pharmacology/Toxicology and Cancer Institute, University of Mississippi Medical Center, Jackson, MS USA

**Keywords:** Exosome, microRNA, miR-10b, Invasion

## Abstract

**Introduction:**

Exosomes are 30-100 nm membrane vesicles of endocytic origin, mediating diverse biological functions including tumor cell invasion, cell-cell communication and antigen presentation through transfer of proteins, mRNAs and microRNAs. Recent evidence suggests that microRNAs can be released through ceramide-dependent secretory machinery regulated by neutral sphingomyelinase 2 (nSMase2) enzyme encoded by the *smpd3* gene that triggers exosome secretion. However, whether exosome-mediated microRNA transfer plays any role in cell invasion remains poorly understood. Thus, the aim of this study was to identify the exosomal microRNAs involved in breast cancer invasion.

**Methods:**

The expression level of endogenous and exosomal miRNAs were examined by real time PCR and the expression level of target proteins were detected by western blot. Scanning electron and confocal microscopy were used to characterize exosomes and to study its uptake and transfer. Luciferase reporter plasmids and its mutant were used to confirm direct targeting. Furthermore, the functional significance of exosomal miR-10b was estimated by invasion assay.

**Results:**

In this study, we demonstrate that microRNA carrying exosomes can be transferred among different cell lines through direct uptake. miR-10b is highly expressed in metastatic breast cancer MDA-MB-231 cells as compared to non-metastatic breast cancer cells or non-malignant breast cells; it is actively secreted into medium via exosomes. In particular, nSMase2 or ceramide promotes the exosome-mediated miR-10b secretion whereas ceramide inhibitor suppresses this secretion. Moreover, upon uptake, miR-10b can suppress the protein level of its target genes such as HOXD10 and KLF4, indicating its functional significance. Finally, treatment with exosomes derived from MDA-MB-231 cells could induce the invasion ability of non-malignant HMLE cells.

**Conclusion:**

Together, our results suggest that a set of specific microRNAs may play an important role in modulating tumor microenvironment through exosomes. Thus, a better understanding of this process may aid in the development of novel therapeutic agents.

**Electronic supplementary material:**

The online version of this article (doi:10.1186/1476-4598-13-256) contains supplementary material, which is available to authorized users.

## Introduction

The biological functions in higher organisms are executed by intercellular communication through complex assemblies of cells, the action of which must be highly coordinated. This cell-cell communication is also a vital aspect for development of various diseases including cancer. It has been demonstrated that the process of cellular communication is achieved by autocrine, paracrine or direct cell to cell contact. There is increasing evidence suggesting that cells may also communicate via other mechanisms in addition to these known methods, including exchange of cellular fragments, membranes or specialized organelles like microvesicles which until recently has been regarded as cellular debris. The two major forms of vesicles are ectosomes and exosomes. Ectosomes emerge from cellular membrane while the exosomes are of endosomal origin[1].

Exosomes are nanovesicles with diameter ranging from 30-120 nm; they are derived from endocytic compartments and are released by many cell types including cancer cells. Exosome biogenesis is a complex and controlled process that begins with the inward budding of cellular membrane to form an early endosome. After the endosome formation, cytoplasmic content is internalized into small vesicles, i.e., exosomes. When exosomes are present inside the endosome, the endosome forms a multivesicular body (MVB) which later on fuses with the cellular membrane to release the exosomes[2]. Exosome cargo includes a variety of biomolecules ranging from lipids, proteins to nucleic acids that include mRNA, microRNA and DNA. In addition to their size and morphology, exosomes can be identified by virtue of their unique protein and lipid composition. This includes raft-lipids such as cholesterol, sphingolipids, ceramide and glycerophospolipids with long and saturated fatty-acyl chains. Due to their endosomal origin, exosomes from different cell types contains common proteins such as Rab GTPases, Annexins, flotillin, Alix, TSG101, hsp70 and hsp90, integrins and tetraspanins (CD63, CD9, CD81 and CD82). Some of these proteins can be used as marker proteins to identify and characterize exosomes. These exosomal marker proteins includes Alix, flotillin, TSG101 and CD63[3]. Recent independent studies have confirmed the idea that these cell-derived vesicles carry and deliver microRNAs to recipient cells in vitro[4–6]. Also, the presence of circulating microRNAs in the blood of cancer patients has raised the possibility that they may serve as a novel diagnostic marker[7]. For example, microRNA profiling on serum from breast cancer patients found that seven microRNAs (miR-10b, miR-21, miR-125b, miR-145, miR-155 miR-191 and miR-382) had different expression patterns in serum of breast cancer patients compared to healthy controls[8]. In particular, the overexpression of miR-21, miR-10b, and miR-19a can be utilized as serum biomarkers for aggressive breast cancer and inflammatory breast cancer as they are associated with the acquisition of malignant characteristics like increased tumor cell proliferation, migration, invasion, dissemination, and metastasis[9]. In another case involving 89 patients with primary breast cancer (n =59) and metastatic disease (n =30), and 29 healthy women, breast cancer-associated miR-10b, miR-34a, miR-141 and miR-155 were elevated in the blood of breast cancer patients and are associated with tumor progression[10]. Although, the role of exosomal miRNAs in cancer progression and metastasis is still poorly understood, emerging evidence suggests cancer-secreted exosomal microRNAs are emerging as mediators of cancer progression and metastasis. For example, Zhou et al., demonstrated that miR-105 is a potent regulator of migration through targeting the tight junction protein ZO-1 and is characteristically expressed and secreted by metastatic breast cancer cells[11]. Overexpression of miR-105 in nonmetastatic breast cancer cells can induce metastasis, whereas inhibition of miR-105 in highly metastatic tumors has the opposite effect[11]. Another study showed that exosomal miR-210 which is secreted by metastatic cancer cells, can enhance angiogenesis after its uptake by endothelial cells through suppression of specific target genes[12]. Also, exosomal miRNAs can directly interact with proteins like Toll-like receptors (TLRs) to drive an immune response and to activate nuclear factor-kB (NF-kB) pathway that can help in tumor generation, development and immune surveillance escape promoting chronic inflammation[13]. These findings indicate that the horizontal transfer of exosomal microRNAs from cancer cells to non-malignant cells can modify the microenviromental niche for their own advantage.

Although it becomes evident now that microRNAs can be carried in exosomes and transported in the circulating system, it remains largely unknown whether these microRNAs have any function. In this study, we present evidence that exosomes can be transferred among different cell lines through direct uptake and in process delivers the microRNAs to the recipient cells. Upon uptake, microRNAs can still reduce the protein level of its target genes, indicating its functional significance. We found that miR-10b derived from MDA-MB-231 cells can be transferred to non-malignant HMLE cells via exosomes. Moreover, this transferred miR-10b can promote invasion in HMLE cells in part by targeting a known miR-10b target, HOXD10[14].

## Materials and methods

### Reagents

HMLE cells were a generous gift from Dr. Robert Weinberg (The Massachusetts Institute of Technology). Primary antibodies were obtained as follows: monoclonal anti-β-actin and anti-HOXD10 from Sigma-Aldrich (Allentown, PA); anti-KLF4 from Cell Signaling Technology (Beverly, MA). Secondary antibodies conjugated with IRDye 800CW or IRDye 680 were purchased from LI-COR Biosciences (Lincoln, NE, USA). Luciferase reporters, pMIR-D10UTR for HOXD10 3′UTR (#19117) and HOXD10 mut 3′ UTR (#19116), were purchased from Addgene (Cambridge MA). PCR primers and FAM-miR-10b were purchased from IDT (Coralville, IA).

### Cell culture

The human MCF-7 and MDA-MB-231 cells were obtained from ATCC (Manassas, VA, USA) and cultured in RPMI-1640 with 10% FBS (Sigma, St. Louis, MO). MCF-10A and HMLE cells were cultured in DMEM/F12K supplemented with growth factor hEGF, hydrocortisone, insulin and 100 units of penicillin/ml and 100 mg of streptomycin/ml. HEK-293T cells were cultured in Dulbecco’s modified Eagle’s medium (DMEM) (Cambrex) supplemented with 10% FBS. All media contained 2 mM glutamine, 100 units of penicillin/ml and 100 mg of streptomycin/ml. Cells were incubated at 37°C and supplemented with 5% CO_2_ in a humidified chamber. For experiments, cells were grown in medium with exosome-free serum. Therefore, the serum added to the cell culture medium was depleted of exosomes by ultracentrifugation at 120,000 × g overnight (16 h) at 4°C, followed by passing it through 0.2 micron filter prior to use.

### Exosome isolation and labeling

Cells for exosome isolation from culture medium were maintained in respective medium with exosome-free FBS which was prepared by centrifugations to remove existing exosomes. Exosomes were isolated from cell culture medium collected after 48 h by differential centrifugations as described by Raposo and colleagues with a small modification[15]. Briefly, the collected culture mixture was centrifuged at 300 × g for 10 min, followed by 2000 × g for 20 min. The supernatant was further filtered by a 0.22-μM filter (Millipore). The obtained medium was centrifugated at 100,000 × g for 60 min at 4°C to pellet exosomes. The supernatant was discarded without disturbing the pellet which was then washed with a large volume of PBS, ultracentrifugated and finally resuspended again in PBS. Purified exosomes were labeled with PKH26 red fluorescent labeling kit (Sigma) as per manufacturer’s protocol.

### Expression vectors

The high fidelity enzyme Phusion was used to amplify respective DNA fragments by PCR to make these constructs. To construct the GFP-CD63 fusion, we first amplified copGFP with primers copGFP-R1-5.1 and copGFP-CD63-3.1; CD63 with primers copGFP-CD63-5.1 and CD63-pCDH-PU-Not1-3.1. These two fragments were then cloned into pCDH-PU (SBI) at EcoR I and Not I site by cold fusion method (SBI). To clone miR-10b, a genomic DNA fragment (~500 bp) carrying miR-10b precursor was amplified from human genomic DNA with miR-10b-5.1 and miR-10b-3.1. The PCR fragment was first cloned into PCR8 (Life Technology) and then this fragment was migrated into pCDH-CMV-copGFP (SBI). SMPD3 expression vector was cloned by the same PCR amplification approach using primers SMPD3-R1-5.1 and SMPD3-Not1-3.1 and then cloned into pCDH-CMV-myc-EF1-GFP-PU at EcoR I and Not I site by cold fusion method. For cloning the KLF4 plus UTR, we used primers KLF4-Myc-R1-5.1 and KLF4-UTR-Not1-3.1, and then cloned into the pCDH-PU (SBI) at EcoR I and Not I restriction site. The sequences of all PCR products were verified by DNA sequencing.

### Transfection

Cells were transfected with miR-10b mimic using RNAfectin reagent or with plasmid DNA using DNAfectin (both from Applied Biological Materials, Vancouver, Canada) following the manufacturer’s protocol.

### Confocal microscopy

Confocal microscopy was carried out by using the Leica confocal microscope at the imaging facility of Southern Illinois University School of Medicine or Nikon C1 confocal microscope at Imaging Core at Center for Psychiatric Neuroscience of UMMC.

### Scanning electron microscopy (SEM)

An aliquot of exosome suspension was loaded on to a carbon-coated electron microscopy grid. The sample was fixed with 2% glutaraldehyde and 2% paraformaldehyde in 0.1 M sodium cacodylate buffer at pH 7.3. After two washes in distilled H_2_O, the samples were stained with 2% methylamine tungstate for 45 s and air-dried. Finally the samples were observed in a Zeiss scanning electron microscope.

### RNA preparation and RT-PCR

For RT-PCR, we isolated total RNA using Trizol reagent (Invitrogen, Carlsbad, CA) as per the manufacturer’s protocol and used 0.5 μg RNA to synthesize cDNA by SuperScriptase III (Invitrogen) with random primers. Finally, the resultant cDNA was used for PCR reactions. PCR annealing temperature varied depending on the primers used. To detect miR-10b, we used the polymerase A method, followed by SYBR Green qPCR as described previously[16]. RNU1 and 5s RNA were used as an internal control.

### Western blotting

Cells were harvested and protein was extracted from cells as previously described[17]. The protein concentration was determined using a protein assay kit (Bio-Rad, Hercules, CA, USA) and samples were separated in SDS polyacrylamide gels, with various concentrations depending on the molecular weight of the protein under investigation. After probing with a primary antibody, the membrane was incubated with a secondary antibody labeled with either IRDye 800CW or IRDye 680. Finally, signal intensity was determined using the Odyssey Infrared Imaging System (LI-COR Biosciences, Lincoln, NE, USA).

### Invasion assay

To determine whether miR-10b can confer invasion ability through exosome transfer, we overexpressed miR-10b in MDA-MB-231 cells and then isolated exosomes from the conditioned medium. The isolated exosomes were mixed with HMLE cells and incubated for 24 h before subject to invasion assays. Specifically, matrigel chambers (BD Biosciences) were used to determine the invasiveness per the manufacturer’s protocol as described previously[18]. In brief, cells were harvested, resuspended in serum-free medium, and then transferred to the hydrated Matrigel chambers (∼25,000 cells per well). The chambers were then incubated for 24 h in culture medium with 10% FBS in the bottom chambers before examination. The cells on the upper surface were scraped and washed away, whereas the invaded cells on the lower surface were fixed and stained with 0.05% crystal violet for 2 h. Finally, invaded cells were counted under a microscope and the relative number was calculated.

### Luciferase reporter assay

Luciferase assays were carried out in HMLE cells. Cells were transfected with firefly luciferase reporters (pMIR-D10UTR for HOXD10 3′UTR and HOXD10 mut 3′ UTR) (400 ng) and 2 ng of the pRL-SV40 renilla luciferase construct in 12-well plates. Then they were incubated with appropriate exosomes for 24 h and finally harvested and lysed for luciferase assays. Luciferase assays were performed using a luciferase assay kit (Promega) according to the manufacturer’s protocol. Renilla luciferase was used for normalization.

### Preparation of the lentiviral particles and infection

Lentiviral packaging was carried out in HEK-293T cells using a packaging system from System Biosciences as per the manufacturer’s protocol, as described previously[19]. For a large scale preparation the viral particles were concentrated by centrifugation. The final titer was at least 1 × 10^8^. For infection, exponentially growing cells were mixed with viral particles in the presence of polybrene (0.8 mg/ml) in a six-well plate at a multiplicity of infection of 1-3.

### Primers for cloning and detection

SMPD3-R1-5.1 CCATGGAGGCCCGAATTCTGGTTTTGTACACGACCCCC

SMPD3-Not1-3.1 TCGCAGATCCTTGCGGCCGCTCCGGACGGTCTATGCCTCC

miR-10b-5.1 GGTTAATAAAGCCGCCATCC

miR-10b-3.1 CTTCTGGAGAGGAAAAGCTG

copGFP-R1-5.1 ATTCTAGAGCTAGCGAATTCGCTAGACGCCACCATGGAGA

copGFP-CD63-5.1 CCGGCTCCACCGGATCTCGCGCGGTGGAAGGAGGAATGAA

copGFP-CD63-3.1 TTCATTCCTCCTTCCACCGCGCGAGATCCGGTGGAGCCGG

KLF4-Myc-R1-5.1 CCATGGAGGCCCGAATTCTGAGGCAGCCACCTGGCGAG

KLF4-UTR-Not1-3.1 TCGCAGATCCTTGCGGCCGCTTAATTCTCACCTTGAGTATG

### Statistical analysis

Data are presented as mean ± SE; the Student’s *t*-test was used for assessing the difference between individual groups and *P* ≤ 0.05 was considered statistically significant.

## Results

### Characterization of exosomes released from cancer cells and their transfer from donor to recipient cells

**Figure 1:**
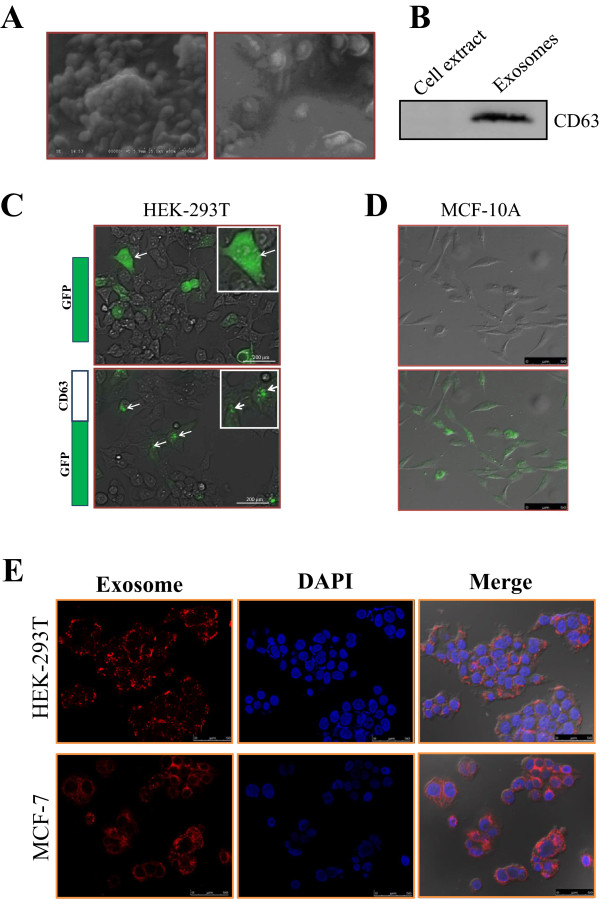
**Characterization of exosome transfer. A**, Electron micrograph of exosomes isolated from MDA-MB-231 conditioned medium, revealing the typical morphology and size (30-100 nm). **B**, Western blot showing presence of CD63 protein in exosome fraction from the same MDA-MB-231 conditioned medium. **C**, HEK-293T cells expressing CD63-GFP fusion protein compared to GFP alone. **D**, Cellular uptake of exosomes isolated from HEK-293T cells expressing the CD63-GFP fusion protein by MCF-10A cells. Image was captured 24 h after addition of exosomes to the culture. **E**, Intercellular trafficking of exosomes among different cell lines by isolated exosomes labeled with PKH26 dye. Shown here are for uptake in MCF-7 and HEK-293T cell lines. Images were taken 24 h after exosome addition by confocal microscope.

Exosomes can be actively released from a variety of cell types including cancer cells. To determine whether microRNA-carrying exosomes can be released from breast cancer cells, we incubated three cell lines, i.e., MCF-10A, MCF-7 and MDA-MB-231, in exosome-free medium made from exosome-free FBS. The exosomes in the conditioned media were isolated by a serial centrifugations and filtration. The harvested exosomes were then resuspended in PBS or other agents depending upon further use[20]. To determine the relative purity of isolated exosomes and their morphology, the exosome pellets were resuspended in PBS and then examined by scanning electron microscopy (SEM). Shown in Figure 1A were the exosomes isolated from MDA-MB-231 cells with a uniformly cup-shaped morphology; their size was within the characteristic diameter range of 40-120 nm. To further determine the nature of the isolated exosomes, we used the exosome-specific marker CD63 which has been used to provide a quantification of the exosomes present in cell culture supernatants[21]. As shown in Figure 1B, CD63 was not detected in whole cell lysate but were found in abundance in the isolated exosomal fraction. These results confirmed that the vesicles isolated from the conditional media were the exosomes based on their size and presence of marker protein, CD63.

To examine whether the secreted exosomes can be taken up by recipient cells, we used two pronged approaches. In the first approach, we cloned CD63 along with GFP as a fusion protein in a lentiviral vector and then generated stable cell lines expressing CD63-GFP fusion. The distribution of CD63-GFP was mainly in speckle like structures (Figure 1C), very different from that of GFP alone which was distributed uniformly in the entire cell. Exosomes were then isolated from the HEK 293T cell line stably over expressing the CD63-GFP fusion protein. After addition of the isolated CD63-GFP tagged exosomes to MCF-10A cells and incubation for 24 h, we examined them under a confocal microscope. We found that almost all of these cells were GFP positive with a similar speckle like structures (Figure 1D). In the second approach, the isolated exosomes from MDA-MB-231 cells were labeled with PKH26 dye (red), washed thoroughly and then added to MCF-7 and HEK-293T cells. The uptake of the labeled exosomes by recipient cells was visualized under confocal microscope. Again, almost all recipient cells revealed red signal (Figure 1E). These results suggest that the uptake of exosomes by these cells is every efficient.

### Quantitative RT-PCR profiling for intracellular and extracellular microRNAs

Having demonstrated that exosomes are released from breast cancer cells and then taken by various types of cells, we determined what kind of microRNAs are secreted via exosomes. We chose metastatic MDA-MB-231 and non-malignant HMLE cells for comparison. We profiled the cancer-related microRNAs secreted in the conditioned media as well as the endogenous level in these two cell lines by qRT-PCR (Additional file1: Table S1 and S2). Among a total of 144 cancer-related microRNAs, the endogenous miR-10b (i.e., from inside the cell) was remarkably and most highly expressed in MDA-MB-231 cells whereas its expression was extremely low in HMLE cells, indicating that high expression of miR-10b is a distinct feature of microRNA profile of MDA-MB-231 cells. Importantly, similar expression pattern was observed in the extracellular (i.e., exosomal) fraction for miR-10b, indicating that miR-10b can be actively secreted by MDA-MB-231 cells but little was detected in HMLE conditioned medium. Other top five intracellular and extracellular microRNAs whose expression was higher in MDA-MB-231 cells as compared to HMLE cells were listed in Tables 1 and2. Since miR-10b has significantly high expression both at intracellular and extracellular level in MDA-MB-231 cells as compared to HMLE cells and it has been shown to plays a vital role in breast cancer invasion and metastasis[14], we focused on miR-10b in the following experiments.

**Figure 2:**
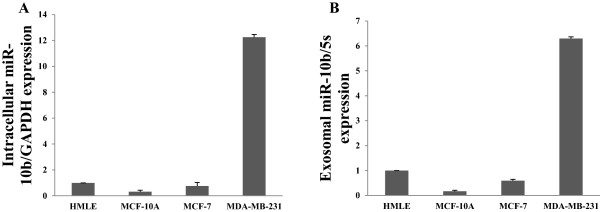
**Upregulation of miR-10 in MDA-MB-231 cells.** Detection of differential expression of intracellular **(A)** and extracellular **(B)** miR-10b in among HMLE, MCF-10A, MCF-7 and MDA-MB-231 cell, as determined by qRT-PCR.

Next, we examined the intracellular and extracellular level of miR-10b in different breast cancer cells ranging from epithelial to metastatic by real time PCR. This includes MCF-10A and MCF-7 cell lines in addition to HMLE and MDA-MB-231 cells. The results indicate that the level of miR-10b is highest both at endogenous and secretory level in metastatic MDA-MB-231 cells as compared to epithelial like HMLE or MCF-10A as well as non-metastatic MCF-7 cells (Figure 2A and B).

**Table 1:** **Top 5 intracellular miRNAs overexpressed in MDA-MB-231 cells as compared to HMLE cells**

S.no	miRNA	Fold expression (SD)
1	miR-10b	13.21 ± 2.14
2	miR-218	12.68 ± 2.04
3	miR-10a	9.53 ± 2.09
4	miR-99a	9.09 ± 1.07
5	miR-142-3p	9.04 ± 0.58

**Table 2:** **Top 5 extracellular miRNAs overexpressed in MDA-MB-231 cells as compared to HMLE**

S.no	miRNA	Fold expression (SD)
1	miR-10b	5.02 ± 0.68
2	miR-32	5.00 ± 4
3	miR-138	4.29 ± 0.04
4	miR-7e	4.13 ± 2.73
5	miR-106b	2.92 ± 0.57

### Secretion of miR-10b is regulated in a ceramide-dependent manner

**Figure 3:**
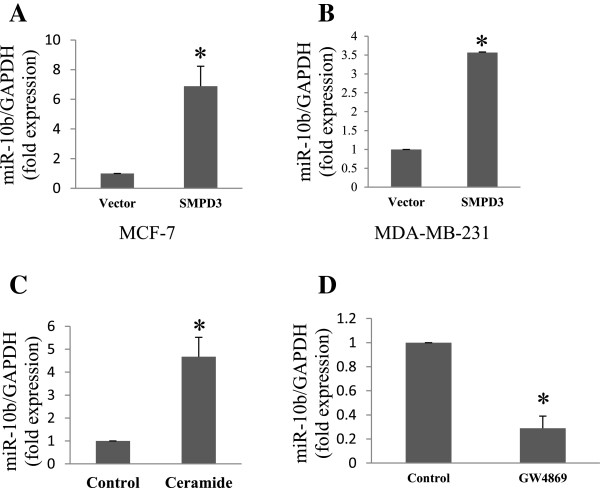
**Regulation of exosomal miR-10b secretin by ceramide biosynthesis pathway. A**, Detection of miR-10b from conditioned medium of MCF-7 cells stably expressing *smpd3* and vector control by qRT-PCR. **B**, Detection of miR-10b from conditioned medium of MDA-MB-231 cells stably expressing *smpd3* and vector control by qRT-PCR. **C**, Secretion of miR-10b is enhanced by the treatment with ceramide in MDA-MB-231 cells. **D**, Suppression of miR-10b secretion by ceramide biosynthesis inhibitor (GW4869) in MDA-MB-231 cells.

There have been various studies on the biogenesis and release of exosomes. For example, Trajkovic *et al*., reported that release of exosomes is independent of the endosomal sorting complex (ESCRT) machinery and is regulated by the sphingolipid ceramide[22]. Further, it has been shown that the secretion of microRNAs is controlled by neutral sphingomyelinase 2 (nSMase2), which is known as a rate-limiting enzyme of ceramide biosynthesis[23]. To determine whether ceramide biosynthesis regulates exosomal miR-10b secretion in breast cancer cells, we cloned the *smpd3* gene coding for nSMase2 in a lentiviral vector. qRT-PCR analysis revealed that the level of secreted miR-10b was significantly higher in both MCF-7 and MDA-MB-231 cells overexpressing *smpd3* than in vector control cells (Figure 3A and B). To further confirm the role of ceramide on miR-10b release, MDA-MB-231 cells were treated with 2 μM ceramide for 48 h. We detected an elevated level of secreted miR-10b (Figure 3C), suggesting that ceramide promotes the secretion of this microRNA. In contrast, when MDA-MB-231 cells were treated with a known ceramide inhibitor, GW4869 at 5 μM concentration for 48 h, there was a significant inhibition in the miR-10b level as compared to vehicle control (Figure 3D). Collectively, these results support the notion that secretion of miR-10b is regulated in a ceramide-dependent manner.

### Transfer of miR-10b from donor cells to the recipient cells through exosomes

**Figure 4:**
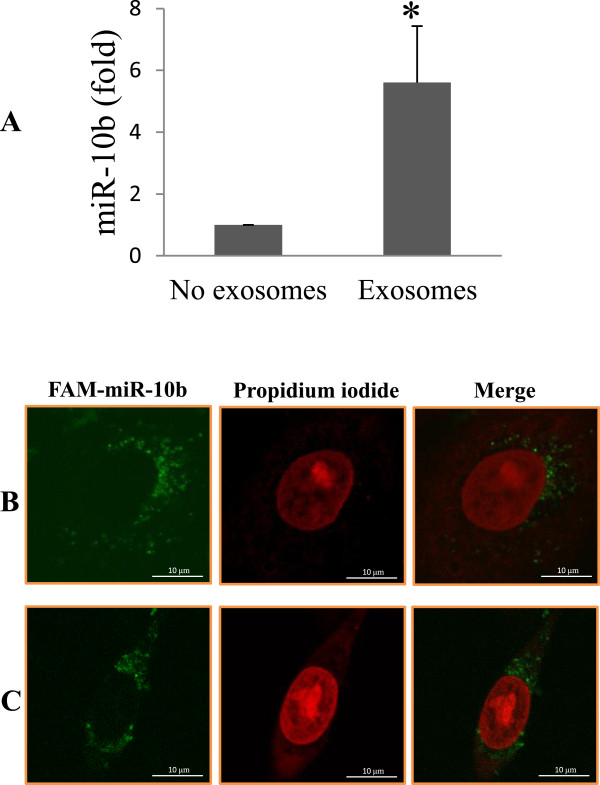
**Uptake of miR-10b by HMLE cells. A**, Exosomal miR-10b was isolated from MDA-MB-231 cells and then added to HMLE cell culture. The control received the exosome-free medium. **B**, Detection of FAM-miR-10b in the exosome-treated HMLE cells. HMLE cells were co-cultured with MDA-MB-231 cells that had been transfected with FAM-labeled-miR-10b using transwell without direct contact. Twenty-four hours after co-culture, HMLE cells were fixed in 4% paraformaldehyde, and analyzed by fluorescent microscope. Green signals indicate FAM-miR-10b while nuclear counterstaining was performed using propidium iodide (PI). **C**, The FAM-miR-10b carrying exosomes were first isolated from the same MDA-MB-231 cells that had been transfected with FAM-labeled-miR-10b and then were added to HMLE culture, fixed and visualized by microscopy. FAM-miR-10b signals (green) were spotted around the nucleus similar to what was seen in B.

To determine whether released microRNAs such as miR-10b can be taken up by various types of cells, we isolated exosomes from MDA-MB-231 cells and then incubated them with HMLE cells. The uptake of miR-10b by HMLE cells was determined by qRT-PCR after exosome treatment. We found a 5-fold increase in the endogenous level of miR-10b in HMLE cells treated with exosomes derived from MDA-MB-231 cells as compared to control. In addition, to visualize the transport of extracellular miR-10b from MDA-MB-231 cells into HMLE cells, MDA-MB-231 cells were transfected with FAM labeled miR-10b and co-cultured with HMLE cells, using transwell. FAM-miR-10b transfected MDA-MB-231 cells were seeded on the transwell membrane whereas HMLE cells were seeded at the bottom well so that they were not directly contacted. We were able to detect the FAM-miR-10b green signal in the cytoplasm of HMLE cells by confocal microscopy 24 h after co-culture (Figure 4B). To further confirm that the transferred miR-10b was derived from exosomal microRNA, we isolated the exosomes from culture medium of MDA-MB-231 cells transfected with FAM-labeled miR-10b. The isolated exosomes were then added to HMLE cells. Again, the miR-10b signal was detected by confocal microscopy in the cytoplasm of HMLE cells 24 h later (Figure 4C) and the signal intensity was similar to what have been observed in Figure 4B. These results strongly suggest that extracellular miR-10b derived from MDA-MB-231 cells can be transferred into HMLE cells and that the horizontal transfer of microRNAs occurs through exosomes among various types of cells.

### Exosomal miR-10b is functional inside the recipient cells and can induce invasion ability in HMLE cells

Having demonstrated that miR-10b can be transferred through exosomes from one cell to another, we next investigated whether miR-10b can still remain functional inside the recipient cell. It has been reported that the miR-10b can induce tumor invasion and initiate metastasis in breast cancer by targeting HOXD10[14] and promotes migration and invasion through direct binding with 3′-UTR of KLF4 in human esophageal cancer cells[24]. To test the functionality of the exosomal miR-10b in recipient cells, we cloned miR-10b in lentiviral vector and generated cells stably expressing miR-10b in MDA-MB-231. We next checked the endogenous and secretory level of miR-10b by real time PCR which confirmed the overexpression of miR-10b by over 15 folds inside the cell and more importantly, the secretory level of miR-10b increased by more than 40 folds when compared to the vector control (Additional file1: Figure S1). For functional assay experiments we used the exosomes isolated from these two stable cells (vector and miR-10b) as they significantly differ in their miR-10b level. Enhanced miR-10b secretion in MDA-MB-231 stable cell line is in consistent with a previously published report that overexpression of microRNA inside the cell increases the extracellular secretion[23].

**Figure 5:**
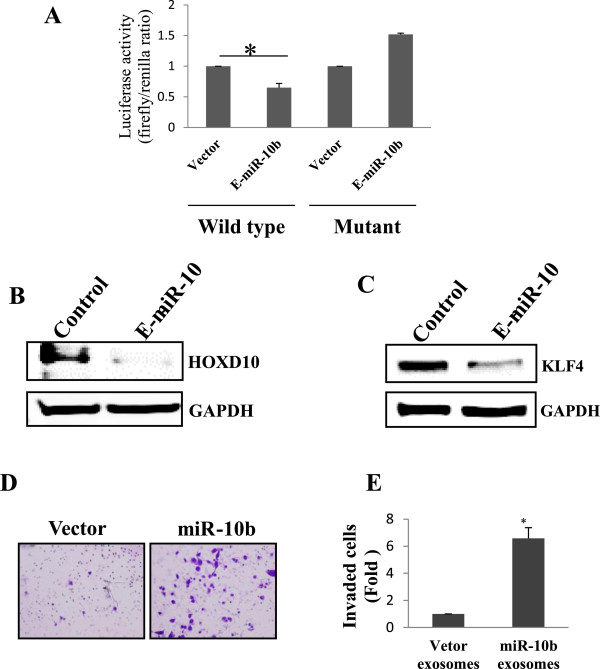
**Exosomal miR-10b promotes cell invasion by targeting HOXD10. A**, Exosomal miR-10b (E-miR-10b) suppresses the luciferase activity of the luciferase reporter carrying HOXD10 3′-UTR. Both wild-type (UTR-WT) or mutant (UTR-mut) reporters were introduced into HMLE cells by transfection and then incubated with the miR-10b-enriched exosomes or control. **B**, Suppression of HOXD10 protein in HMLE cells by exosomal miR-10b. Protein extract was prepared 48 h after addition of exosomal miR1-10b to the HMLE culture. **C**, Suppression of KLF4 in HEK-293T cells transfected with in KLF4 plus 3′-UTR. **D**, Suppression of cell invasion in HMLE cells by exosomal miR-10b. **E**, Quantitative analysis of data obtained from D.

It has been reported that miR-10b overexpression does not cause degradation of HOXD10 mRNA, but it reduces the HOXD10 protein level, suggesting a translational repression mechanism. This silencing action depends on single miR-10b cognate binding site within the 3′-UTR region of HOXD10 gene as a mutant with four nucleotide substitution fails to reduce the activity of a luciferase reporter system[14]. To determine that exosomal miR-10b can have a similar effect on the luciferase activity, we performed luciferase assays with the wild type human HOXD10 3′-UTR; the mutant HOXD10 3′-UTR that has a substitution of 4 nucleotides within the miR-10b binding site, from TCGTAATGCAGGGTA to TCGTAATGCACCCAA as a control. These two constructs were introduced into HMLE cells along with renilla luciferase construct (for normalization purpose). The transfected cells were then treated with miR-10b enriched exosomes or the control exosomes. The miR-10 enriched exosomes could reduce the activity of luciferase reporter gene in wild type 3′-UTR reporter but not in the mutant 3′-UTR reporter as compared to control exosomes (Figure 5A).

To demonstrate the suppression of the endogenous HOXD10 by exosomal miR-10b, exosomes were collected from culture medium of MDA-MB-231 cell line derived from the stably overexpressing miR-10b and the vector control after 48 h and added to HMLE cells. After 24 h of exosome treatment, the protein level of HOXD10 was determined by western blot which indicates down regulation of this protein in cells treated with miR-10b enriched exosomes as compared to vector control (Figure 5B).

Another known target of miR-10b is KLF4[24]. Since HMLE cells express little KLF4, we cloned KLF4 coding region plus its 3′-UTR and the protein was tagged with Myc at the N-terminus. This construct was then transiently transfected into HEK-293T cells. Again, the miR-10b enriched exosomes along with control exosomes were added to HEK-293T cells with KLF4 construct. After 24 h of transfection, there was an obvious decrease in the KLF4 protein level in cells treated with miR-10b enriched exosomes as compared to vector control (Figure 5C). This provides additional evidence that secreted miR-10b can still remain functional after uptake in the recipient cells.

Finally, given that suppression of HOXD10 increases cell invasion[14], we determined whether miR-10b carried on exosomes can induce invasion ability of HMLE cells by a transwell invasion assay. After 24 h incubation of HMLE cells with the enriched exosomes derived from miR-10b and vector control, there was a significant increased invasion ability as compared to the vector control (Figure 5D). For example, invasion ability for miR-10b exosomes was over a 6-fold higher than for vector exosomes (Figure 5E), suggesting that exosomal miR-10b remains functional inside the recipient HMLE cells after uptake and can induce the invasion ability by silencing miR-10b targets such as HOXD10 involved in cell invasion.

## Discussion

Accumulating evidence indicates that microRNAs play a key role in tumor progression and metastasis and they can be stably present in the circulating system. Thus, these microRNAs have been explored as cancer biomarkers[25, 26]. Furthermore, a specific set of exosomal microRNAs may modulate the tumor microenvironment[27]. However, it remains largely unknown as to whether and how exosomal microRNAs play a role in cancer development and progression. This study identifies miR-10b as an exosomal microRNA that promotes cell invasion in HMLE cells by targeting HOXD10, suggesting that invasive tumor cells may use exosomal microRNAs as a means for their advance.

We first confirmed that exosome secretion from tumor cells is an active process which is regulated by the ceramide biosynthesis pathway[22]. In consistent with the previous reports[22, 23], we showed that overexpression of *smpd3* gene or exogenous ceramide increases the amount of exosomal microRNAs; on the other hand, treatment with GW4869 results in a reduced secretion of exosomal microRNAs. We also showed that overexpression of miR-10b in MDA-MB-231 cells leads to an increase of exosomal miR-10b in the conditioned medium, suggesting that the intracellular level of microRNAs affects the amount of corresponding exosomal microRNAs.

When exosomes are released to the medium, they can be taken up by recipient cells which can be the same type or a different type of cells. Thus, we isolated exosomes from either HEK-29T or MDA-MB-231 cells and then examined their uptake by MCF-10A, HEK-239T or MCF-7 cells. Overall, the uptake seems very efficient. For example, using the GFP-CD63 fusion reporter, we found that all of MCF-10A cells reveal GFP signal after incubation with exsomes isolated from HEK-293T cell transfected with GFP-CD63 (Figure 1D). Similarly, the uptake is also high in HEK-239T or MCF-7 cells when isolated exosomes were stained with PKH26 dye (Figure 1D and E). These results suggest that microRNA transfer via exosomes among different types of cells occur readily.

Increasing evidence indicates that secreted microRNAs can have a significant impact in a variety of physiological and pathological conditions. Yang *et al*., showed that exosomal miR-223, secreted from macrophages can promote the invasion of breast cancer cells via Mef2c-β-catenin pathway[28]. Exosomal microRNAs derived from K562 cells release the miR-17-92 cluster which target integrin α5 in HUVECs upon uptake, and enhance endothelial cell migration and tube formation[29]. Of interest, miR-21 and miR-29a secreted from tumors can bind as ligands to toll-like receptors in immune cells and trigger prometastatic inflammatory responses that may promote tumor growth and metastasis[13]. Since cancer cells secrete the microRNAs through exosomes into the circulatory system, this feature can be utilized as a new approach for the detection of various malignancies. For instance, serum miR-141 levels have been reported to help distinguish patients suffering from prostate cancer from healthy controls[30]. Our study provides evidence that there is a significant difference in the level of exosomal microRNAs secreted by different breast cancer cell lines. For instance, the level of exosomal miR-10b is much higher in metastatic breast cancer MDA-MB-231 cells than non-metastatic MCF-7 cells. Importantly, this exosomal miR-10b which is actively secreted by MDA-MB-231 cells can be taken up by non-malignant epithelial breast cells to promote their cell invasion.

Various studies have demonstrated that several microRNAs are involved in breast cancer progression and metastasis. These include miR-9[31], miR-10b[14], miR-21[32], miR-29a[33], miR-155[34], miR-200a[35], miR-374a[36] and several other microRNAs. Metastasis is a multistep process which involves epithelial to mesenchymal transition (EMT), intravasation, transport via circulation, extravasation, mesenchymal to epithelial transition (MET), micrometastasis and finally metastasis. One of the major requirement of this complex and multistep process of metastasis is cell to cell communication. The most common mode of cell-cell communication involves two cells in direct contact with each other, communicating via gap junctions. Other methods include paracrine signaling where secretions from one cell have an effect only on cells in the immediate area and endocrine signaling involving hormones that are released into the circulatory system which then reach to the target cell. Our result provides evidence that exosomes are also one of the important mediators of cell-cell communication.

In this regard, exosomal miR-10b derived from metastatic MDA-MB-231 cells is capable of inducing invasion ability upon uptake in non-invasive breast epithelial HMLE cells. It is well known that breast tumors are very heterogeneous, and the ability of tumor cells in the same tumor to invade and progress can vary a lot. Our finding may suggest that less invasive tumor cells can become more invasive by obtaining exosomal microRNAs from the invasive tumor cells in the tumor microenvironment. If this is the case, it would be conceivable that tumor cells can cooperate through exosome-mediated microRNA transfer to advance in the hostile environment, which supports the “game theory” of cancer evolution proposed by Dr. Robert Axelrod[37].

In summary, our work provides another piece of evidence that microRNAs can be transported through exosomes from among different cell types facilitating cell to cell communication. Oncogenic microRNAs like miR-10b, secreted by breast cancer cells, can influence the adjacent and distant normal cells which can lead to outcome in favor of tumor development and progression. Based on these results we envision that targeting exosomal microRNAs may provide an alternative approach for breast cancer intervention.

## Electronic supplementary material

Additional file 1:**Supplementary information.**(PPTX 124 KB)

## Abbreviations

EMT: Mesenchymal transition
MVB: Multivesicular body
MET: Epithelial transition
NSMase2: Neutral sphingomyelinase 2
SEM: Scanning electron microscopy.
