# Supplementary material for: Exosome-mediated transfer of miR-10b promotes cell invasion in breast cancer
Source: Mol Cancer. 2014 Nov 26;13:256. doi: 10.1186/1476-4598-13-256 (PMC4258287; doi:10.1186/1476-4598-13-256)
Supplement: Supplementary file 1 — Additional file 1:Supplementary information.(PPTX 124 KB) [file 12943_2014_1454_MOESM1_ESM.pptx]

## Slide 1
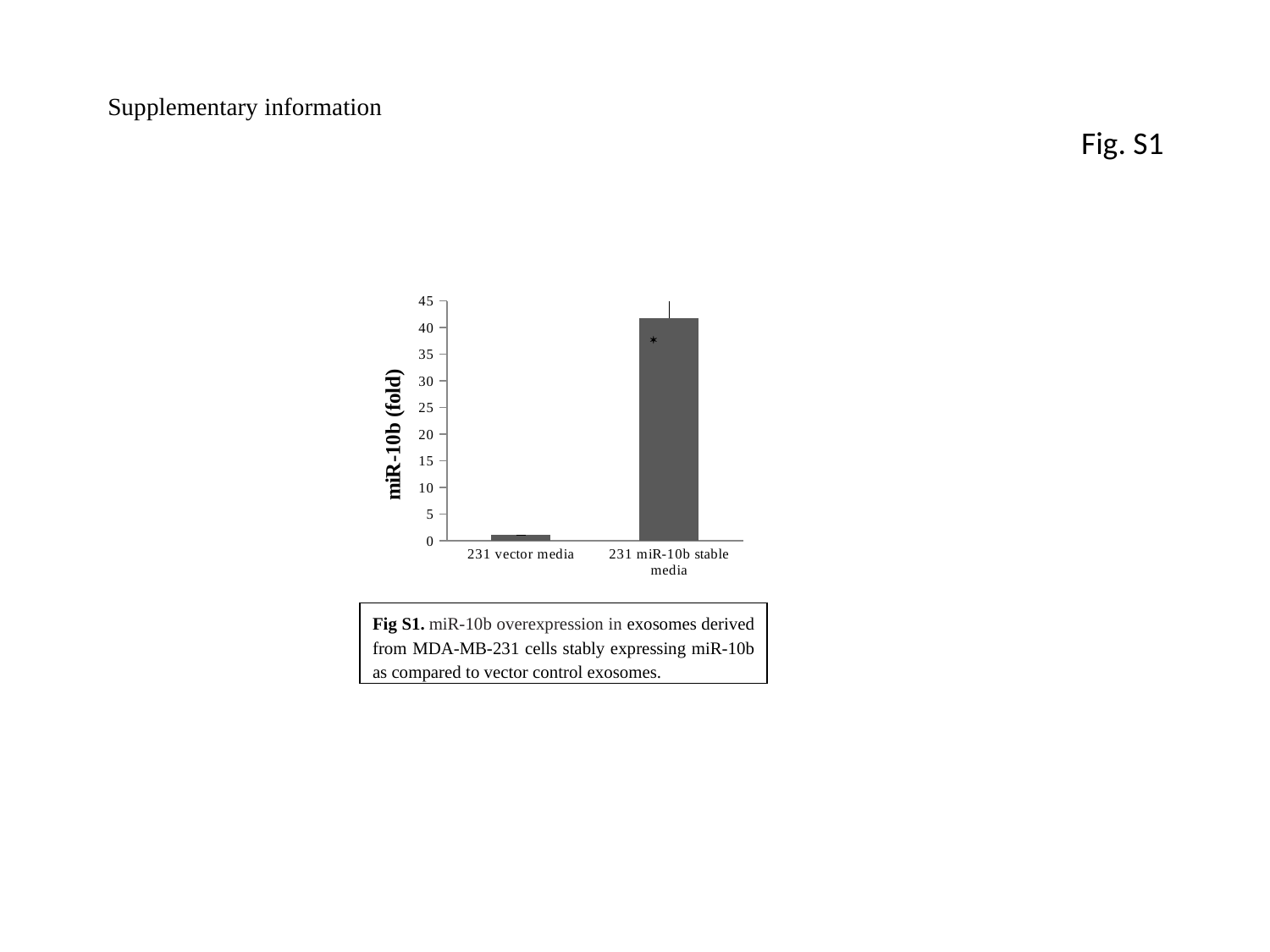

Supplementary information
Fig. S1
### Chart
| Category | miR-10b |
|---|---|
| 231 vector media | 1.0 |
| 231 miR-10b stable media | 41.77 |
Fig S1. miR-10b overexpression in exosomes derived from MDA-MB-231 cells stably expressing miR-10b as compared to vector control exosomes.

## Slide 2
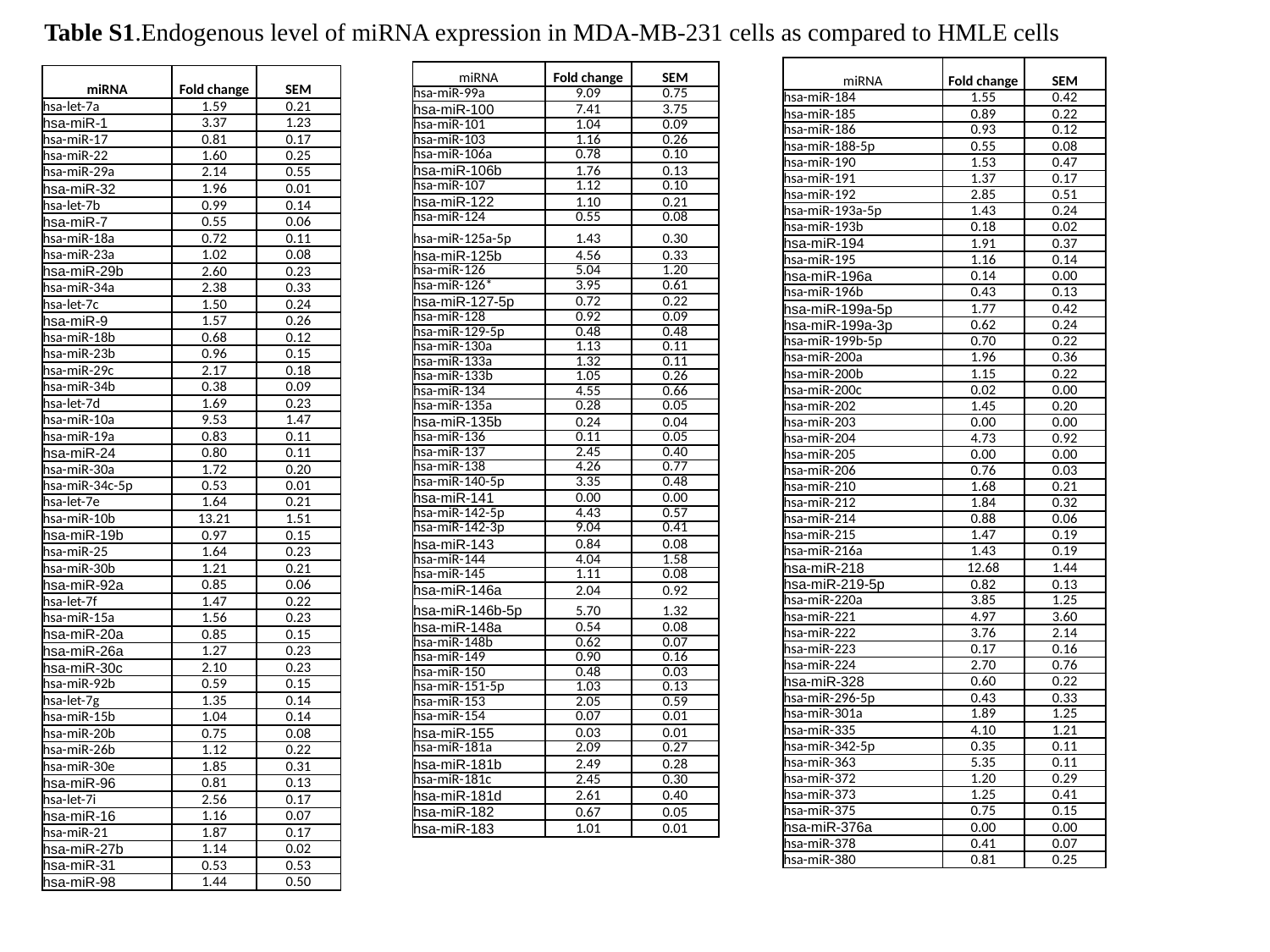

Table S1.Endogenous level of miRNA expression in MDA-MB-231 cells as compared to HMLE cells
| miRNA | Fold change | SEM |
| --- | --- | --- |
| hsa-miR-184 | 1.55 | 0.42 |
| hsa-miR-185 | 0.89 | 0.22 |
| hsa-miR-186 | 0.93 | 0.12 |
| hsa-miR-188-5p | 0.55 | 0.08 |
| hsa-miR-190 | 1.53 | 0.47 |
| hsa-miR-191 | 1.37 | 0.17 |
| hsa-miR-192 | 2.85 | 0.51 |
| hsa-miR-193a-5p | 1.43 | 0.24 |
| hsa-miR-193b | 0.18 | 0.02 |
| hsa-miR-194 | 1.91 | 0.37 |
| hsa-miR-195 | 1.16 | 0.14 |
| hsa-miR-196a | 0.14 | 0.00 |
| hsa-miR-196b | 0.43 | 0.13 |
| hsa-miR-199a-5p | 1.77 | 0.42 |
| hsa-miR-199a-3p | 0.62 | 0.24 |
| hsa-miR-199b-5p | 0.70 | 0.22 |
| hsa-miR-200a | 1.96 | 0.36 |
| hsa-miR-200b | 1.15 | 0.22 |
| hsa-miR-200c | 0.02 | 0.00 |
| hsa-miR-202 | 1.45 | 0.20 |
| hsa-miR-203 | 0.00 | 0.00 |
| hsa-miR-204 | 4.73 | 0.92 |
| hsa-miR-205 | 0.00 | 0.00 |
| hsa-miR-206 | 0.76 | 0.03 |
| hsa-miR-210 | 1.68 | 0.21 |
| hsa-miR-212 | 1.84 | 0.32 |
| hsa-miR-214 | 0.88 | 0.06 |
| hsa-miR-215 | 1.47 | 0.19 |
| hsa-miR-216a | 1.43 | 0.19 |
| hsa-miR-218 | 12.68 | 1.44 |
| hsa-miR-219-5p | 0.82 | 0.13 |
| hsa-miR-220a | 3.85 | 1.25 |
| hsa-miR-221 | 4.97 | 3.60 |
| hsa-miR-222 | 3.76 | 2.14 |
| hsa-miR-223 | 0.17 | 0.16 |
| hsa-miR-224 | 2.70 | 0.76 |
| hsa-miR-328 | 0.60 | 0.22 |
| hsa-miR-296-5p | 0.43 | 0.33 |
| hsa-miR-301a | 1.89 | 1.25 |
| hsa-miR-335 | 4.10 | 1.21 |
| hsa-miR-342-5p | 0.35 | 0.11 |
| hsa-miR-363 | 5.35 | 0.11 |
| hsa-miR-372 | 1.20 | 0.29 |
| hsa-miR-373 | 1.25 | 0.41 |
| hsa-miR-375 | 0.75 | 0.15 |
| hsa-miR-376a | 0.00 | 0.00 |
| hsa-miR-378 | 0.41 | 0.07 |
| hsa-miR-380 | 0.81 | 0.25 |
| miRNA | Fold change | SEM |
| --- | --- | --- |
| hsa-miR-99a | 9.09 | 0.75 |
| hsa-miR-100 | 7.41 | 3.75 |
| hsa-miR-101 | 1.04 | 0.09 |
| hsa-miR-103 | 1.16 | 0.26 |
| hsa-miR-106a | 0.78 | 0.10 |
| hsa-miR-106b | 1.76 | 0.13 |
| hsa-miR-107 | 1.12 | 0.10 |
| hsa-miR-122 | 1.10 | 0.21 |
| hsa-miR-124 | 0.55 | 0.08 |
| hsa-miR-125a-5p | 1.43 | 0.30 |
| hsa-miR-125b | 4.56 | 0.33 |
| hsa-miR-126 | 5.04 | 1.20 |
| hsa-miR-126\* | 3.95 | 0.61 |
| hsa-miR-127-5p | 0.72 | 0.22 |
| hsa-miR-128 | 0.92 | 0.09 |
| hsa-miR-129-5p | 0.48 | 0.48 |
| hsa-miR-130a | 1.13 | 0.11 |
| hsa-miR-133a | 1.32 | 0.11 |
| hsa-miR-133b | 1.05 | 0.26 |
| hsa-miR-134 | 4.55 | 0.66 |
| hsa-miR-135a | 0.28 | 0.05 |
| hsa-miR-135b | 0.24 | 0.04 |
| hsa-miR-136 | 0.11 | 0.05 |
| hsa-miR-137 | 2.45 | 0.40 |
| hsa-miR-138 | 4.26 | 0.77 |
| hsa-miR-140-5p | 3.35 | 0.48 |
| hsa-miR-141 | 0.00 | 0.00 |
| hsa-miR-142-5p | 4.43 | 0.57 |
| hsa-miR-142-3p | 9.04 | 0.41 |
| hsa-miR-143 | 0.84 | 0.08 |
| hsa-miR-144 | 4.04 | 1.58 |
| hsa-miR-145 | 1.11 | 0.08 |
| hsa-miR-146a | 2.04 | 0.92 |
| hsa-miR-146b-5p | 5.70 | 1.32 |
| hsa-miR-148a | 0.54 | 0.08 |
| hsa-miR-148b | 0.62 | 0.07 |
| hsa-miR-149 | 0.90 | 0.16 |
| hsa-miR-150 | 0.48 | 0.03 |
| hsa-miR-151-5p | 1.03 | 0.13 |
| hsa-miR-153 | 2.05 | 0.59 |
| hsa-miR-154 | 0.07 | 0.01 |
| hsa-miR-155 | 0.03 | 0.01 |
| hsa-miR-181a | 2.09 | 0.27 |
| hsa-miR-181b | 2.49 | 0.28 |
| hsa-miR-181c | 2.45 | 0.30 |
| hsa-miR-181d | 2.61 | 0.40 |
| hsa-miR-182 | 0.67 | 0.05 |
| hsa-miR-183 | 1.01 | 0.01 |
| miRNA | Fold change | SEM |
| --- | --- | --- |
| hsa-let-7a | 1.59 | 0.21 |
| hsa-miR-1 | 3.37 | 1.23 |
| hsa-miR-17 | 0.81 | 0.17 |
| hsa-miR-22 | 1.60 | 0.25 |
| hsa-miR-29a | 2.14 | 0.55 |
| hsa-miR-32 | 1.96 | 0.01 |
| hsa-let-7b | 0.99 | 0.14 |
| hsa-miR-7 | 0.55 | 0.06 |
| hsa-miR-18a | 0.72 | 0.11 |
| hsa-miR-23a | 1.02 | 0.08 |
| hsa-miR-29b | 2.60 | 0.23 |
| hsa-miR-34a | 2.38 | 0.33 |
| hsa-let-7c | 1.50 | 0.24 |
| hsa-miR-9 | 1.57 | 0.26 |
| hsa-miR-18b | 0.68 | 0.12 |
| hsa-miR-23b | 0.96 | 0.15 |
| hsa-miR-29c | 2.17 | 0.18 |
| hsa-miR-34b | 0.38 | 0.09 |
| hsa-let-7d | 1.69 | 0.23 |
| hsa-miR-10a | 9.53 | 1.47 |
| hsa-miR-19a | 0.83 | 0.11 |
| hsa-miR-24 | 0.80 | 0.11 |
| hsa-miR-30a | 1.72 | 0.20 |
| hsa-miR-34c-5p | 0.53 | 0.01 |
| hsa-let-7e | 1.64 | 0.21 |
| hsa-miR-10b | 13.21 | 1.51 |
| hsa-miR-19b | 0.97 | 0.15 |
| hsa-miR-25 | 1.64 | 0.23 |
| hsa-miR-30b | 1.21 | 0.21 |
| hsa-miR-92a | 0.85 | 0.06 |
| hsa-let-7f | 1.47 | 0.22 |
| hsa-miR-15a | 1.56 | 0.23 |
| hsa-miR-20a | 0.85 | 0.15 |
| hsa-miR-26a | 1.27 | 0.23 |
| hsa-miR-30c | 2.10 | 0.23 |
| hsa-miR-92b | 0.59 | 0.15 |
| hsa-let-7g | 1.35 | 0.14 |
| hsa-miR-15b | 1.04 | 0.14 |
| hsa-miR-20b | 0.75 | 0.08 |
| hsa-miR-26b | 1.12 | 0.22 |
| hsa-miR-30e | 1.85 | 0.31 |
| hsa-miR-96 | 0.81 | 0.13 |
| hsa-let-7i | 2.56 | 0.17 |
| hsa-miR-16 | 1.16 | 0.07 |
| hsa-miR-21 | 1.87 | 0.17 |
| hsa-miR-27b | 1.14 | 0.02 |
| hsa-miR-31 | 0.53 | 0.53 |
| hsa-miR-98 | 1.44 | 0.50 |

## Slide 3
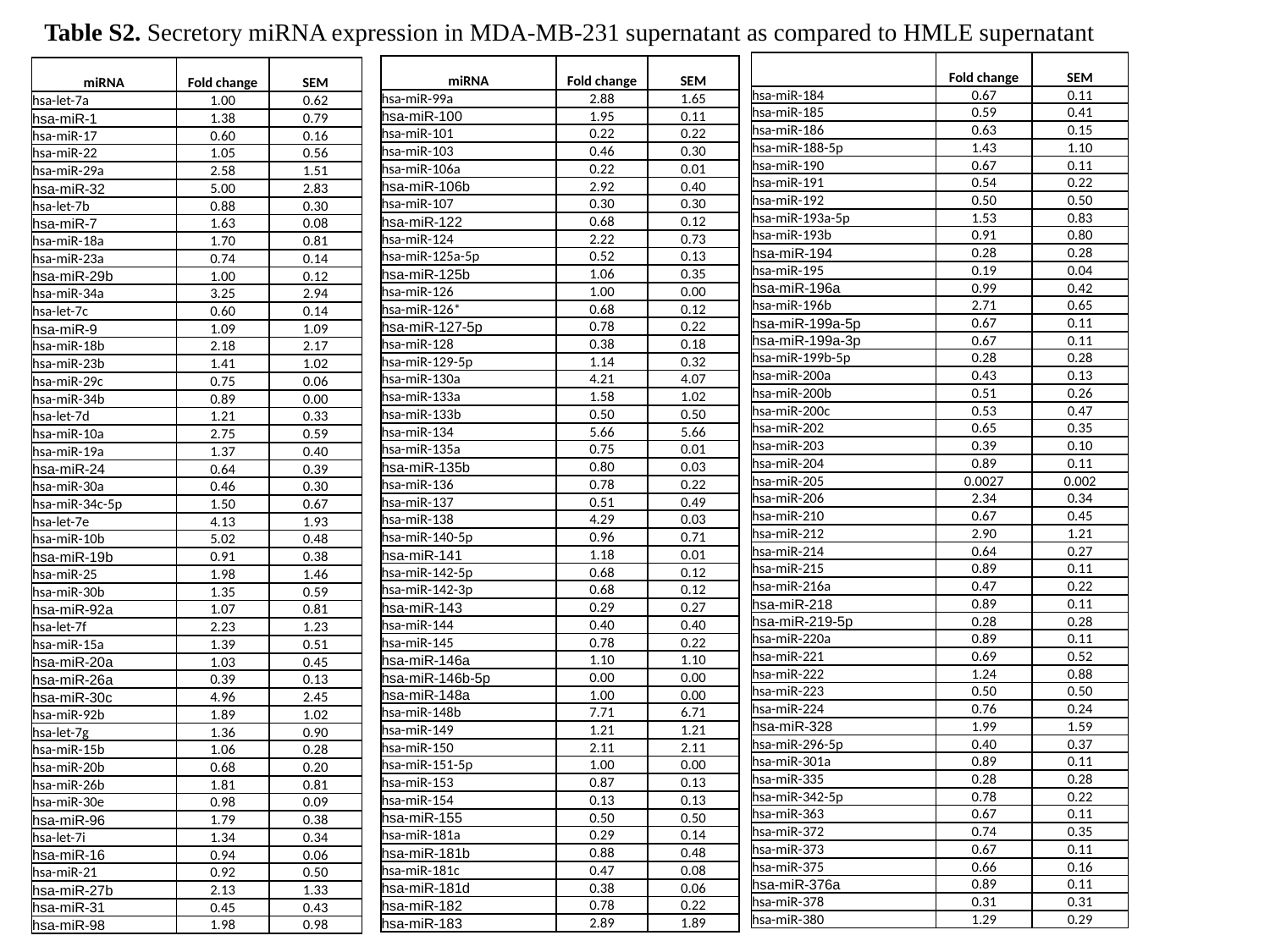

Table S2. Secretory miRNA expression in MDA-MB-231 supernatant as compared to HMLE supernatant
| | Fold change | SEM |
| --- | --- | --- |
| hsa-miR-184 | 0.67 | 0.11 |
| hsa-miR-185 | 0.59 | 0.41 |
| hsa-miR-186 | 0.63 | 0.15 |
| hsa-miR-188-5p | 1.43 | 1.10 |
| hsa-miR-190 | 0.67 | 0.11 |
| hsa-miR-191 | 0.54 | 0.22 |
| hsa-miR-192 | 0.50 | 0.50 |
| hsa-miR-193a-5p | 1.53 | 0.83 |
| hsa-miR-193b | 0.91 | 0.80 |
| hsa-miR-194 | 0.28 | 0.28 |
| hsa-miR-195 | 0.19 | 0.04 |
| hsa-miR-196a | 0.99 | 0.42 |
| hsa-miR-196b | 2.71 | 0.65 |
| hsa-miR-199a-5p | 0.67 | 0.11 |
| hsa-miR-199a-3p | 0.67 | 0.11 |
| hsa-miR-199b-5p | 0.28 | 0.28 |
| hsa-miR-200a | 0.43 | 0.13 |
| hsa-miR-200b | 0.51 | 0.26 |
| hsa-miR-200c | 0.53 | 0.47 |
| hsa-miR-202 | 0.65 | 0.35 |
| hsa-miR-203 | 0.39 | 0.10 |
| hsa-miR-204 | 0.89 | 0.11 |
| hsa-miR-205 | 0.0027 | 0.002 |
| hsa-miR-206 | 2.34 | 0.34 |
| hsa-miR-210 | 0.67 | 0.45 |
| hsa-miR-212 | 2.90 | 1.21 |
| hsa-miR-214 | 0.64 | 0.27 |
| hsa-miR-215 | 0.89 | 0.11 |
| hsa-miR-216a | 0.47 | 0.22 |
| hsa-miR-218 | 0.89 | 0.11 |
| hsa-miR-219-5p | 0.28 | 0.28 |
| hsa-miR-220a | 0.89 | 0.11 |
| hsa-miR-221 | 0.69 | 0.52 |
| hsa-miR-222 | 1.24 | 0.88 |
| hsa-miR-223 | 0.50 | 0.50 |
| hsa-miR-224 | 0.76 | 0.24 |
| hsa-miR-328 | 1.99 | 1.59 |
| hsa-miR-296-5p | 0.40 | 0.37 |
| hsa-miR-301a | 0.89 | 0.11 |
| hsa-miR-335 | 0.28 | 0.28 |
| hsa-miR-342-5p | 0.78 | 0.22 |
| hsa-miR-363 | 0.67 | 0.11 |
| hsa-miR-372 | 0.74 | 0.35 |
| hsa-miR-373 | 0.67 | 0.11 |
| hsa-miR-375 | 0.66 | 0.16 |
| hsa-miR-376a | 0.89 | 0.11 |
| hsa-miR-378 | 0.31 | 0.31 |
| hsa-miR-380 | 1.29 | 0.29 |
| miRNA | Fold change | SEM |
| --- | --- | --- |
| hsa-miR-99a | 2.88 | 1.65 |
| hsa-miR-100 | 1.95 | 0.11 |
| hsa-miR-101 | 0.22 | 0.22 |
| hsa-miR-103 | 0.46 | 0.30 |
| hsa-miR-106a | 0.22 | 0.01 |
| hsa-miR-106b | 2.92 | 0.40 |
| hsa-miR-107 | 0.30 | 0.30 |
| hsa-miR-122 | 0.68 | 0.12 |
| hsa-miR-124 | 2.22 | 0.73 |
| hsa-miR-125a-5p | 0.52 | 0.13 |
| hsa-miR-125b | 1.06 | 0.35 |
| hsa-miR-126 | 1.00 | 0.00 |
| hsa-miR-126\* | 0.68 | 0.12 |
| hsa-miR-127-5p | 0.78 | 0.22 |
| hsa-miR-128 | 0.38 | 0.18 |
| hsa-miR-129-5p | 1.14 | 0.32 |
| hsa-miR-130a | 4.21 | 4.07 |
| hsa-miR-133a | 1.58 | 1.02 |
| hsa-miR-133b | 0.50 | 0.50 |
| hsa-miR-134 | 5.66 | 5.66 |
| hsa-miR-135a | 0.75 | 0.01 |
| hsa-miR-135b | 0.80 | 0.03 |
| hsa-miR-136 | 0.78 | 0.22 |
| hsa-miR-137 | 0.51 | 0.49 |
| hsa-miR-138 | 4.29 | 0.03 |
| hsa-miR-140-5p | 0.96 | 0.71 |
| hsa-miR-141 | 1.18 | 0.01 |
| hsa-miR-142-5p | 0.68 | 0.12 |
| hsa-miR-142-3p | 0.68 | 0.12 |
| hsa-miR-143 | 0.29 | 0.27 |
| hsa-miR-144 | 0.40 | 0.40 |
| hsa-miR-145 | 0.78 | 0.22 |
| hsa-miR-146a | 1.10 | 1.10 |
| hsa-miR-146b-5p | 0.00 | 0.00 |
| hsa-miR-148a | 1.00 | 0.00 |
| hsa-miR-148b | 7.71 | 6.71 |
| hsa-miR-149 | 1.21 | 1.21 |
| hsa-miR-150 | 2.11 | 2.11 |
| hsa-miR-151-5p | 1.00 | 0.00 |
| hsa-miR-153 | 0.87 | 0.13 |
| hsa-miR-154 | 0.13 | 0.13 |
| hsa-miR-155 | 0.50 | 0.50 |
| hsa-miR-181a | 0.29 | 0.14 |
| hsa-miR-181b | 0.88 | 0.48 |
| hsa-miR-181c | 0.47 | 0.08 |
| hsa-miR-181d | 0.38 | 0.06 |
| hsa-miR-182 | 0.78 | 0.22 |
| hsa-miR-183 | 2.89 | 1.89 |
| miRNA | Fold change | SEM |
| --- | --- | --- |
| hsa-let-7a | 1.00 | 0.62 |
| hsa-miR-1 | 1.38 | 0.79 |
| hsa-miR-17 | 0.60 | 0.16 |
| hsa-miR-22 | 1.05 | 0.56 |
| hsa-miR-29a | 2.58 | 1.51 |
| hsa-miR-32 | 5.00 | 2.83 |
| hsa-let-7b | 0.88 | 0.30 |
| hsa-miR-7 | 1.63 | 0.08 |
| hsa-miR-18a | 1.70 | 0.81 |
| hsa-miR-23a | 0.74 | 0.14 |
| hsa-miR-29b | 1.00 | 0.12 |
| hsa-miR-34a | 3.25 | 2.94 |
| hsa-let-7c | 0.60 | 0.14 |
| hsa-miR-9 | 1.09 | 1.09 |
| hsa-miR-18b | 2.18 | 2.17 |
| hsa-miR-23b | 1.41 | 1.02 |
| hsa-miR-29c | 0.75 | 0.06 |
| hsa-miR-34b | 0.89 | 0.00 |
| hsa-let-7d | 1.21 | 0.33 |
| hsa-miR-10a | 2.75 | 0.59 |
| hsa-miR-19a | 1.37 | 0.40 |
| hsa-miR-24 | 0.64 | 0.39 |
| hsa-miR-30a | 0.46 | 0.30 |
| hsa-miR-34c-5p | 1.50 | 0.67 |
| hsa-let-7e | 4.13 | 1.93 |
| hsa-miR-10b | 5.02 | 0.48 |
| hsa-miR-19b | 0.91 | 0.38 |
| hsa-miR-25 | 1.98 | 1.46 |
| hsa-miR-30b | 1.35 | 0.59 |
| hsa-miR-92a | 1.07 | 0.81 |
| hsa-let-7f | 2.23 | 1.23 |
| hsa-miR-15a | 1.39 | 0.51 |
| hsa-miR-20a | 1.03 | 0.45 |
| hsa-miR-26a | 0.39 | 0.13 |
| hsa-miR-30c | 4.96 | 2.45 |
| hsa-miR-92b | 1.89 | 1.02 |
| hsa-let-7g | 1.36 | 0.90 |
| hsa-miR-15b | 1.06 | 0.28 |
| hsa-miR-20b | 0.68 | 0.20 |
| hsa-miR-26b | 1.81 | 0.81 |
| hsa-miR-30e | 0.98 | 0.09 |
| hsa-miR-96 | 1.79 | 0.38 |
| hsa-let-7i | 1.34 | 0.34 |
| hsa-miR-16 | 0.94 | 0.06 |
| hsa-miR-21 | 0.92 | 0.50 |
| hsa-miR-27b | 2.13 | 1.33 |
| hsa-miR-31 | 0.45 | 0.43 |
| hsa-miR-98 | 1.98 | 0.98 |
